# Supplementary figures and images for: The modified MIDA-Score predicts mid-term outcomes after interventional therapy of functional mitral regurgitation
Source: PLoS One. 2020 Jul 22;15(7):e0236265. doi: 10.1371/journal.pone.0236265 (PMC7375538; doi:10.1371/journal.pone.0236265)

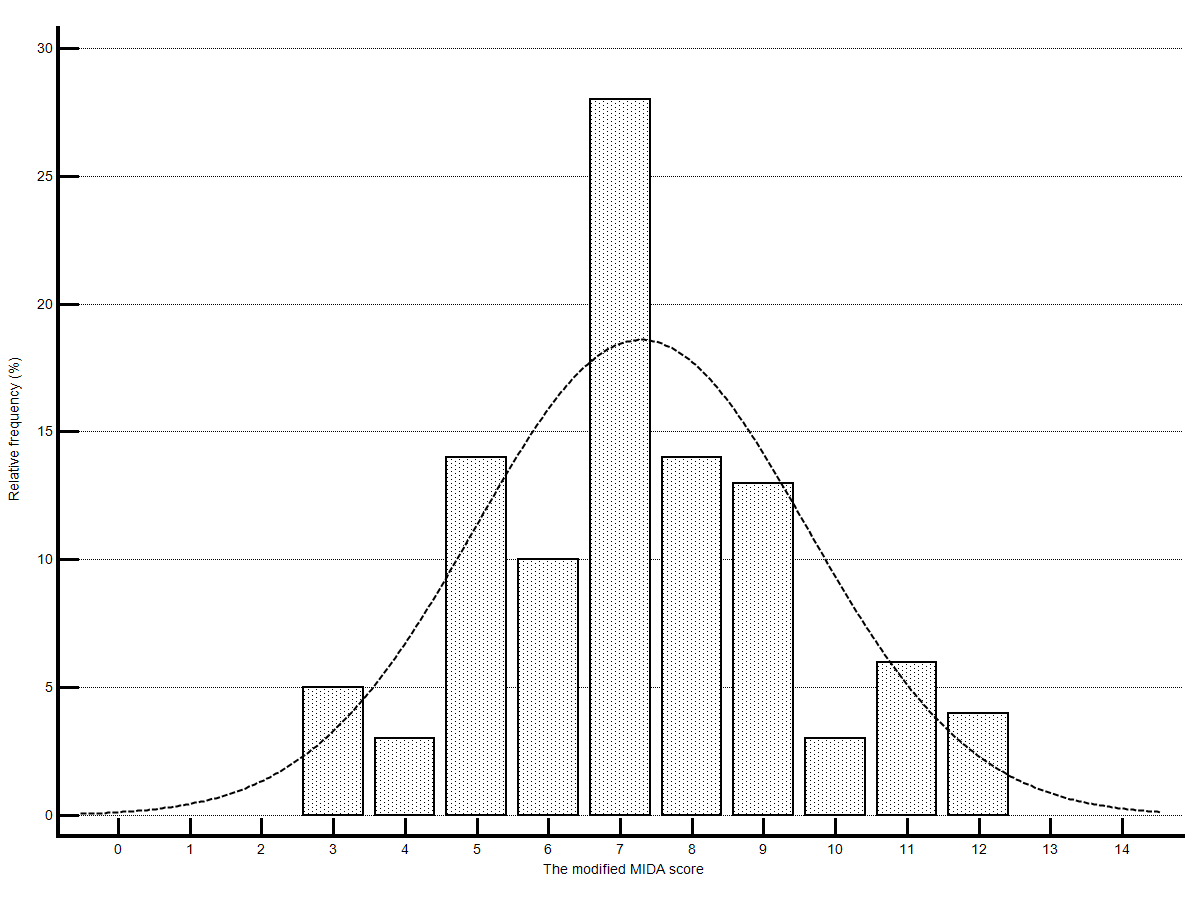

Supplement: S1 Fig — (TIF) [file pone.0236265.s001.tif]

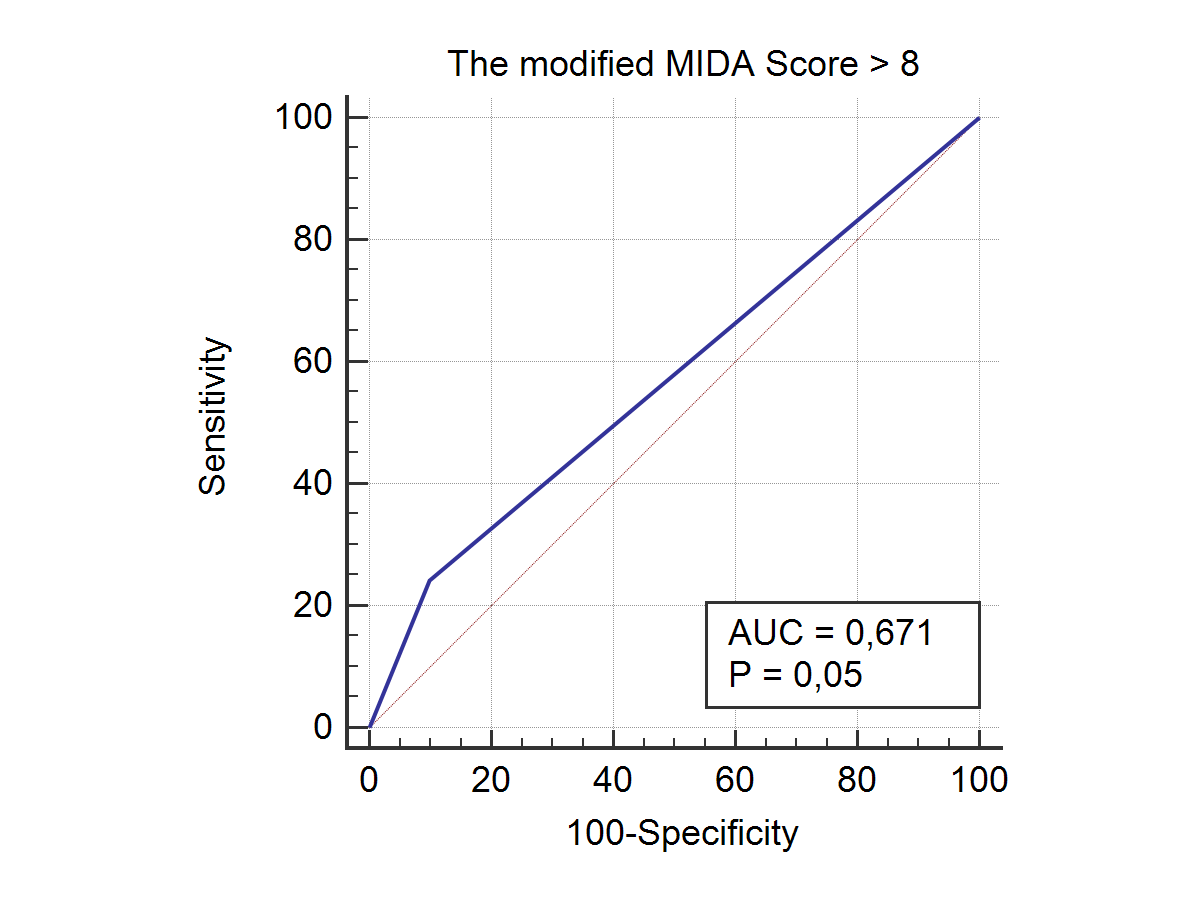

Supplement: S2 Fig — (TIF) [file pone.0236265.s002.tif]

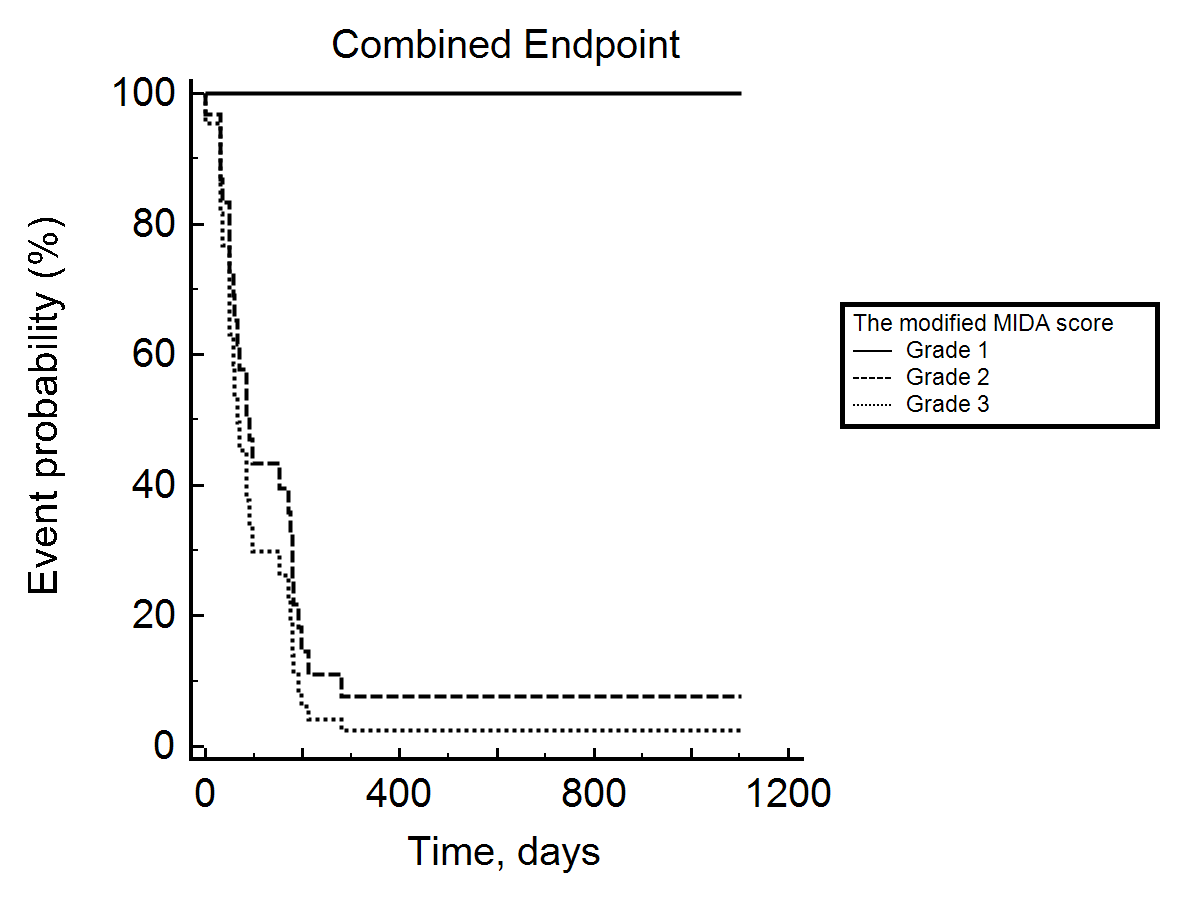

Supplement: S3 Fig — (TIF) [file pone.0236265.s003.tif]

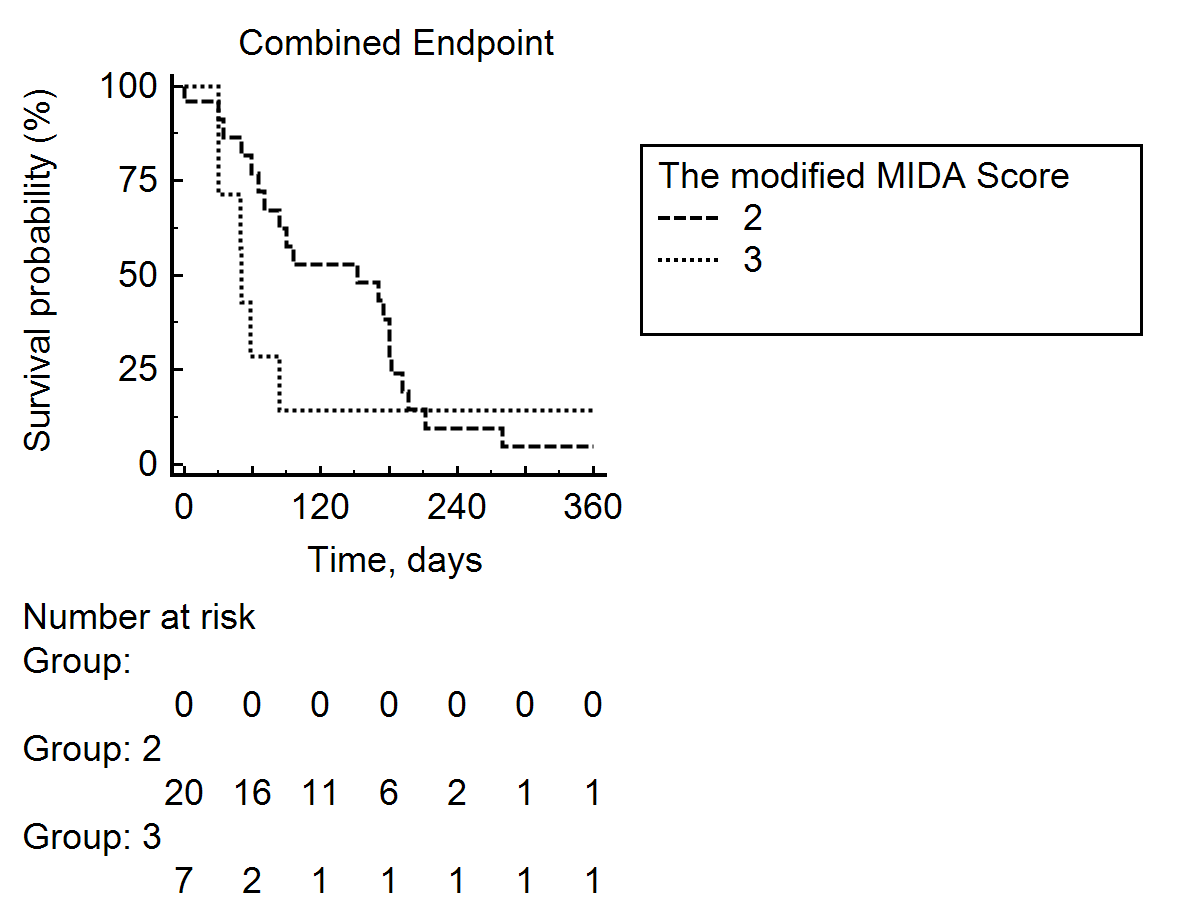

Supplement: S4 Fig — (TIF) [file pone.0236265.s004.tif]
