# Supplementary material for: The modified MIDA-Score predicts mid-term outcomes after interventional therapy of functional mitral regurgitation
Source: PLoS One. 2020 Jul 22;15(7):e0236265. doi: 10.1371/journal.pone.0236265 (PMC7375538; doi:10.1371/journal.pone.0236265)
Supplement: Attachment — Submitted filename: Response to Reviewers.docx [file pone.0236265.s005.docx]

To the

Editorial Board of

PLOS ONE

Universitätsklinikum Bonn, Sigmund-Freud-Str. 25, 53127 Bonn

Bonn, 23. März 2020

Dear Sirs and Madams,

Herewith we are submitting our revised manuscript entitled "*The modified MIDA-Score predicts mid-term outcomes after interventional therapy of functional mitral regurgitation*" **(PONE-D-19-35111)** for further evaluation of possible publication in ***PLOS ONE.***

We thank the reviewers and the editor for their fair, competent, and valuable evaluation of the present manuscript. We think that the implementation of their comments helped to improve the implication of the manuscript substantially. As suggested by the editor, we answered all comments on a point-by-point basis, which you will find attached below, and marked relevant changes in the manuscript with Track Changes before resubmission.

**Journal Requirements:**

***Point 1: Please ensure that your manuscript meets PLOS ONE's style requirements, including those for file naming.***

We checked and adapted the revised manuscript to PLOS ONE´s style requirements.

***Point 2:***

***a) In ethics statement in the manuscript and in the online submission form, please provide***

***additional information about the patient records used in your retrospective study. Specifically,***

***please ensure that you have discussed whether all data were fully anonymized before you***

***accessed them and/or whether the IRB or ethics committee waived the requirement for informed***

***consent. If patients provided informed written consent to have data from their medical records***

***used in research, please include this information.***

We extended ethical information and added in the materials and methods section, as you recommended.

“The register study was following the Declaration of Helsinki and was authorized by the local ethics committee "Ethics Committee of the Faculty of Medicine at the University of Bonn." All patients signed written informed consent for using their medical records for research and the Bonner registry before the study inclusion. All patient-related data were fully anonymized before using in the study and transferred to the register system. “

***b)*** ***Moreover, please discuss more in detail how the items of the new score were generated.***

We thank the editor for this comment. We have already explained the improvement process of the new scoring system, adapted scoring system, in the results section under the subtitle "A novel scoring system for FMR" in detail (Lines 203-269). The parameters of the modified MIDA-Score are not newly generated. We adapted their cut-offs to patients with functional mitral regurgitation regarding different underlying pathomechanism by reference to ROC analysis of our cohort and mentioned four huge previous meta-analyses in 8864 patients. Moreover, we involved various metanalysis (see references) of functional mitral regurgitation in our development process.

***Point 3: Thank you for including your ethics statement: The register Study was following the***

***Declaration of Helsinki and was authorized by the local ethics committee. All patients signed***

***written informed consent for the Bonner registry.***

***i) Please amend your current ethics statement to include the ** full name ** of the ethics***

***committee/institutional review board(s) that approved your specific study.***

We added the required information and extended our ethics statement in the manuscript.

„The Bonner Mitral Valve Register was following the Declaration of Helsinki and was authorized by the local ethics committee “Ethics Committee of the Faculty of Medicine at the University of Bonn”. All patients signed written informed consent for using their medical records for research before the registration in Bonner Mitral Valve Register Database. All patient-related data were fully anonymized before entry in the Database and analyzed for the study.“

***ii) Once you have amended this/these statement(s) in the Methods section of the manuscript,***

***please add the same text to the “Ethics Statement” field of the submission form (via “Edit***

***Submission”).***

The extended ethics information will be added to the “Ethics Statement” field of the submission form.

***Point 4: We note that you have stated that you will provide repository information for your data at***

***acceptance. Should your manuscript be accepted for publication, we will hold it until you provide***

***the relevant accession numbers or DOIs necessary to access your data. If you wish to make***

***changes to your Data Availability statement, please describe these changes in your cover letter***

***, and we will update your Data Availability statement to reflect the information you provide.***

We don’t want to change our Data Availability statement.

***Point 5: Thank you for stating the following financial disclosure:***

***"NO".***

***a) Please provide an amended Funding Statement that declares *all* the funding or sources***

***of support received during this specific study (whether external or internal to your***

***organization)***

The authors received no specific funding and supportive sources for this work.

***b) Please state what role the funders took in the study. If any authors received a salary from***

***any of your funders, please state which authors and which funder. If the funders had no***

***role, please state: "The funders had no role in study design, data collection and analysis,***

***decision to publish, or preparation of the manuscript."***

 There were no supportive funding and source in this study.

***Reviewers´ comments:***

***Reviewer #1:***

We would like to thank you for your careful review of our manuscript. We have revised our manuscript to incorporate your recommendation as fully as possible. Responses to the specific points are given below.

***Comment 1:*** - ***Check for spelling and grammar (eg line 36 „as“ instead of was, line 91 „life quality“ instead of quality of life, …), professional manuscript editing services is strongly recommended.***

We thank the reviewer for this comment. We changed “life quality” into “quality of life” in line 55-56. We modified the sentence in line 39-40 to avoid ambiguousness.

„Mitral regurgitation (MR) is a common valvular heart disease associated with high mortality and reduced quality of life.“

„Logistic regression analysis showed the modified MIDA score above 9 points to be the strongest predictor for mortality and rehospitalization after TMVR (OR: 3.35, p=0.011). “

***Comment 2: There is not sufficient data in the manuscript to write „enables proper patient selection, adequate therapy decision, and timing” in the conclusion.***

We agree with the reviewer on this point and rewritten as suggested.

In the Abstracts section:

„The modified MIDA score was found to offer a promising early-stage scoring system predicting individual postinterventional prognosis in our patients with FMR underwent TMVR. Further validation in large-scale prospective multicentric studies is warranted.“

In the Conclusion section:

„The modified MIDA Score was found to be a promising early-stage tool predicting individual postinterventional prognosis in our patients with FMR underwent TMVR, which might enable proper patient selection and adequate decision-making. Our preliminary data should be validated by a multicentric study with a huge number of patients. „

***Comment 3: Why was the inclusion set from 01/2014 until 08/2016? FU of 18 months would have allowed also patients from 2017 and 2018. As limited numbers are a major drawback, the authors should consider to enlarge their cohort.***

We thank the reviewer for this comment. We agree that the limited number of patients is a major limitation, and the period of inclusion is somewhat long ago. We included the patients with longer follow-ups and completed clinical and echocardiographic data in this study to make a functional and echocardiographical analysis. Unfortunately, the recent patients had either uncompleted data or short follow-ups.

***Comment 4: „primary endpoint “ is a term which should be reserved for prospective trials. At least as soon as one speaks of primary endpoints also secondary endpoints should be defined.***

We thank the reviewer for this remark. We defined the primary endpoint as a combined endpoint consisting of cardiovascular mortality and rehospitalization due to cardiac decompensation for proper prognostic assessment in patients with FMR. Secondary endpoints were all-cause mortality and rehospitalization separately. We adapted and rewritten related paragraph in the materials and method section.

„In line with MVARC definitions (Mitral valve academic research consortium), we defined the primary endpoint as a combined endpoint consisting of cardiovascular mortality and rehospitalization due to cardiac decompensation. Secondary endpoints were all-cause mortality and rehospitalization.„

***Comment 5: Description of echo assessment in detail does not offer any important informations as we all have to follow the guidelines and standard best practice.***

We appreciate the important comment on this issue. However, we think that our echocardiographical protocols and measurements should be stated in the Methods section for providing complete and transparent data presentation. The modified MIDA-Score consists of three clinical and four echocardiographical parameters, which can be echocardiographically assessed or measured in different ways. Therefore, it is important to show echocardiographical assessments for maintaining the reproducibility of our data. On the other hand, this manuscript can be read by colleagues without echocardiographic advance expertise.

***Comment 6:*** ***Generally spoken it seems a bit arbitrary that a combination of mortality and hospitalisation as endpoint was chosen. Probably the low mortality rate required an additional factor to reach at least a little statistical power. This is legitimate if explained in a clear and structured way. However The authors should think about a larger time frame or about a cooperation with other centers to significantly increase patient numbers.***

We thank the reviewer for this remark. In this study, we showed a prognostic scoring system for functional mitral regurgitation as a novel idea. It may cause rising attention in this field and encourage making further studies. We are planning to make a multicentric study with a huge number of patients as a validation study. We choose a combined endpoint consisting of the critical and clinical relevant parameter to sufficiently represent adverse outcomes in those patients.

**Reviewer # 2:**

We would like to thank you for your careful review of our manuscript. We have revised our manuscript to incorporate your recommendation as fully as possible. Responses to the specific points are given below.

***Comment 1: please add that and state clearly that this the retrospective analysis has a***

***high risk for selection bias.***

We agree with the reviewer on this point and extended the limitation section as recommended.

„ The retrospective nature of this study and targeted patient selection from the Database might be reasons for bias.“

***Comment 2: Line 404, Conclusion section: what is meant with “…primitively…”? Please rephrase.***

The term “primitively” was withdrawn, and the sentence was rewritten, as you suggested. It was used to explain that this novel scoring system is an early-stage score that should be validated.

„The modified MIDA Score was found to be a promising early-stage tool predicting individual postinterventional prognosis in our patients with FMR underwent TMVR, which might enable proper patient selection and adequate decision-making. Our preliminary data should be validated by a multicentric study with a huge number of patients. „

***Comment 3: Line 244: “pressure is often finding in those…” please rephrase to “..pressure is a frequent finding in those….”***

We agree with the reviewer and rewritten as suggested;

„Furthermore, elevated LV enddiastolic filling pressure associated with high right ventricular systolic pressure is a frequent finding in those patients. “

Yours sincerely,

Can Öztürk, MD

Herzzentrum

Universitätsklinikum Bonn

Sigmund-Freud Strasse 25

53105 Bonn

Germany

Tel.:	+49 228-287-16137

Fax:	+49 228-287-19017

E-mail: can.oeztuerk@ukbonn.de
